# crispRdesignR: A Versatile Guide RNA Design Package in R for CRISPR/Cas9 Applications

**DOI:** 10.7150/jgen.41196

**Published:** 2020-05-18

**Authors:** Dylan Beeber, Frédéric JJ Chain

**Affiliations:** Department of Biological Sciences, University of Massachusetts Lowell, 1 University Ave., Lowell, 01852 USA

**Keywords:** gRNA design, genome editing, guide efficiency, CRISPR, GUI, off-target

## Abstract

The success of CRISPR/Cas9 gene editing applications relies on the efficiency of the single guide RNA (sgRNA) used in conjunction with the Cas9 protein. Current sgRNA design software vary in the details they provide on sgRNA sequence efficiency and usually limit organism choice to a list of developer-selected species. The *crispRdesignR* package aims to address these limitations by providing comprehensive sequence features of the generated sgRNAs in a single program, which allows users to predict sgRNA efficiency and design sgRNA sequences for systems that currently do not have optimized efficiency scoring methods. *crispRdesignR* reports extensive information on all designed sgRNA sequences with robust off-target calling and annotation and can be run in a user-friendly graphical interface. The *crispRdesignR* package is implemented in R and has fully editable code for specialized purposes including sgRNA design in user-provided genomes. The package is platform independent and extendable, with its source code and documentation freely available at https://github.com/dylanbeeber/crispRdesignR.

## Introduction

The CRISPR/Cas9 system has attracted attention in recent years for its ability to edit and regulate DNA in a wide variety of organisms and cell types. Using a strand of single guide RNA (sgRNA), the Cas9 protein is able to search a cellular genome and induce double stranded breaks at a target sequence complementary to the sgRNA that can then be modified^1^. However, several sequence features of the sgRNA and surrounding DNA sequence can influence the enzymatic activity of Cas9^2^. Crucially, the genomic DNA must contain a protospacer adjacent motif (PAM) in the region immediately following the 3' end of the target DNA for Cas9 to recognize the sequence^1^. Other sgRNA sequence features of the guide related to the nucleotide composition like homopolymers and self-complementarity can also affect the activity of the sgRNA^2^.

The efficiency of the sgRNA is a major factor in the success of Cas9 gene editing applications^2^. To predict the efficiency of sgRNA sequences, scoring methods have been developed by applying machine learning techniques to CRISPR/Cas9 experimental data^3^,^4^,^5^. These efficiency scoring methods are accurate within the parameters of the experiments they were based on. However, the predictions are not necessarily generalizable to Cas9 applications in all cell types, organisms, and PAMs not included in the efficiency scoring experimental data^6^. At their most predictive, scoring methods have been shown to only explain about 40% of the variation in efficiency for most guides^6^. Known sequence features that decrease sgRNA efficiency are not always considered by scoring models^3^,^4^, which could result in suggesting inactive sgRNAs. Using the scores provided by these machine learning models in conjunction with considering the effects of sequence features on activity can improve overall guide design^7^.

Potential sgRNA sequences that contain a sequence feature not conducive to Cas9 enzymatic activity can be scored highly by efficiency scoring methods that have not been trained on that feature. In order to generate the most active sgRNA, sequence features must be considered alongside efficiency scoring, however current programs designed to identify suitable sgRNAs might not report all sequence features relevant to sgRNA efficiency^7^. Features like sgRNA self-complementarity, presence of homopolymers, and potential off-target effects can drastically affect experimental outcomes and are often not considered by scoring models^3^,^4^. sgRNA sequences that are able to form hairpins with themselves or with other regions of the RNA backbone have been shown to affect sgRNA activity^8^,^9^, and as such is a feature that should generally be avoided. Homopolymers that contain 4 or more consecutive identical base pairs (e.g. GGGG) can decrease cutting activity, and a homopolymer with 4 consecutive T's will be terminated prematurely in systems that utilize RNA polymerase III to create the sgRNA^10^. It is possible for Cas9 to target and cleave DNA sequences with multiple mismatches to the guide RNA resulting in off-target effects^3^. While often problematic for those working with Cas9, these off-target sequences as well as hairpins and homopolymers can be predicted from the sequence features of the guide RNA. Such features are expected to affect activity more consistently across different cell types, organisms and PAMs than specific nucleotide position features^2^.

Current sgRNA design tools are commonly developed in Python and Perl, with several exclusively deployed as web applications only accessible through a browser^11^. A variety of methods are used to search a target genome for off-targets, resulting in search times that can vary from several seconds to several hours depending on factors such as the length of the target genome and the number of sgRNA sequences reported^11^. Importantly, these tools vary drastically in the information that they provide the user, with differences in the number of mismatches that are allowed for target searching and filtering steps that prevent sgRNAs from being reported based off internal criteria^11^.

We have developed the R package *crispRdesignR* to improve upon current sgRNA design software for CRISPR/Cas9 applications by providing all guides that match a customizable PAM sequence within a target region of any genome using the advanced Doench Rule Set 2 predictive model^3^, and by reporting sequence features often missing from other available programs but important in the CRISPR/Cas9 system including the GC content, self-complementarity, presence of homopolymers, and potential off-target effects for each candidate sgRNA. These additional sequence features accompany the sgRNA results as separate output tables, as they are not included in the efficiency scoring model. This information is especially useful for working with non-standard Cas9 applications where the efficiency score may not be reliable. An optional table can be generated that displays supplementary information on where the potential off-target effects occur in a user-selected genome. *crispRdesignR* is a guide RNA design program written in R, allowing easy integration with other R-based workflows. The *crispRdesignR* package can also be utilized with a graphical user interface for easier accessibility to non-bioinformaticians. In addition, the flexibility of this R package allows users to design sgRNAs in uncommon organisms not currently accessible through other design tools by inputting custom genomes and annotation files for analysis, highlighting the versatility of *crispRdesignR*.

## Materials and Methods

### Model Features

The predictive sgRNA efficiency scoring model used in *crispRdesignR* examines the same features as the Doench 2016 model^3^ except for the cut site within the resulting protein, because not every Cas9 target site is located in a protein encoding region. Our program employs a gradient boosted regression model trained on the FC and RES data sets used in Doench Rule Set 2. The FC and RES data sets^3^ contain about 5000 sgRNA sites plus context sequence (30-mer) for a variety of different genes. Ranks for each sgRNA site are calculated from read counts and normalized between 0 and 1, which is used by the gradient boosting algorithm gbm^12^ to predict sgRNA activity. The Doench 2016 scoring method is trained on guide RNA utilizing the 5'NGG3' PAM sequence, however *crispRdesignR* can design guides for any PAM sequence that is 6 base pairs or fewer. When designing guides for custom PAM sequences, *crispRdesignR* does not change the scoring method because many of the sequence features considered by Doench *et al.*^3^ are unrelated to the PAM sequence. It is however important to note that the accuracy score provided is expected to be less accurate when designing sgRNA sequences with custom PAMs.

The presence of specific nucleotides at certain positions in a sgRNA target site can influence the activity of that site. *crispRdesignR* will consider the single and dinucleotides at each position and convert them into features that the machine learning model uses to predict activity. In accordance with the Doench Rule Set 2^3^, *crispRdesignR* accounts for the presence of position-dependent single nucleotides, position-dependent dinucleotides, single nucleotide count, dinucleotide count, GC count, nucleotides that bookend the PAM sequence, and thermodynamic features of the target sequence plus context region (30-mer). As in Doench Rule Set 2, nucleotide features are one-hot encoded, meaning that the presence of a nucleotide in a position is either “off” (0) or “on” (1). This leads to four features for each single nucleotide position (A, C, T, or G) and sixteen features for each dinucleotide position (AA, AC, AG, AT, etc.). One-hot encoding of these features is crucial for accurate machine learning predictions and is made possible by the vtreat package^13^. A position-independent total count of single and dinucleotides is also used. This is simply the number of each specific nucleotide and dinucleotide combination in the 30-mer. Four features counting each single nucleotide and sixteen features counting each dinucleotide are recorded.

The GC count of the target site (20-mer) is taken and converted into a single feature (a number between 0 and 20). However, two additional GC features are taken, one binary variable for if the GC count is above 10 and another for if the GC count is below 10. The two nucleotides that bookend the “GG” of the PAM site are one-hot encoded as a dinucleotide feature. These are the nucleotides at position 25 and 28 of the 30-mer. As with the position-dependent dinucleotide features, these two nucleotides are converted into 16 binary features, one for each possible dinucleotide combination.

Four thermodynamic features are recorded, one for the predicted melting temperature (Tm) of the sgRNA plus context sequence (30-mer), one for the Tm of the five nucleotides upstream from the PAM (positions 20-24), one for the Tm of the eight nucleotides upstream from the previous 5-mer (positions 12-19) and one for the Tm of the five nucleotides upstream from the 8-mer (positions 7-11). The Doench Rule Set 2 uses the Tm_staluc function from biopython to calculate the Tm of these regions, so the function employed by *crispRdesignR* mirrors the Tm_staluc function using thermodynamic data from Allawi and SantaLucia^14^.

### Model Predictions

The model features were used to train a gradient boosted regression model with the R package gbm^12^ on the FC and RES data used by the Doench Rule Set 2. Position-dependent features that contained no variation due to the restrictive PAM site were removed. Other features that showed no impact on the predictive power of the model were also removed. To predict the efficiency of package-generated sgRNA target sequences, the same features collected to design the model are collected for each possible target site. The generated data are then run through the gbm package and return a number from 0 to 1 for each target site, with 0 indicating less activity and 1 indicating greater activity.

### Off-Target Annotation

Users may search any genome that is provided through the BSgenome package^15^. BSgenome also allows users to import custom genomes and DNA sequences from FASTA files using the *forgeBSgenomeDataPkg* command on a seed file that describes the paths to the raw sequence data in FASTA format (more information can be found in the BSgenome documentation). Genome annotation files (.gtf) can be acquired through the Ensembl and BioMart databases or users can upload their own. Larger genomes should be loaded as a compressed .gtf file (.gtf.gz) due to size limitations.

When off-target searching is on, each sgRNA sequence is checked for the presence of possible off-target sequences with up to four mismatches in the 20-mer. Off-target sequences must match the rules of the PAM site or be included in the list of possible 5'NGG3' PAM mismatches made available by Doench *et al.*^3^. Users interested in searching for off-targets with multiple alternate custom PAMs can run the program multiple times with different custom PAM sequences. Off-target sequences that contain 4 mismatches and do not directly match the PAM sequence are not reported by *crispRdesignR* as they are highly unlikely to be active^3^. The *matchPattern*() function available in the package BioStrings^16^ is used to collect data on each possible off-target sequence. *matchPattern*() searches the target genome for matching patterns up to 4 mismatches. Indels are not considered when searching for matches. When searching genomes with many base pairs (e.g. over 1 billion) it is recommended to keep the DNA query sequence under 500 base pairs to keep the search time to several minutes. The *matchPattern*() function is slower than other match finding methods because it does not require the genome to be pre-indexed, which itself takes additional time. However, this method allows users to easily search uploaded custom genomes without prior processing.

The locations of the possible off-target sequences are cross referenced with a user supplied genome annotation file (.gtf) and reports an off-target information table listing each possible off-target along with the sgRNA target site that it matches. *crispRdesignR* reports sgRNA target sequences and other perfect genomic matches in the off-target annotation table so that the user may verify their target location within the genome. The off-target information table lists the sequence type of the off-target, as well as the gene ID, gene name, and exon number. Any mismatched bases between the off-target and original guide sequence are highlighted in red within this table. A cutting frequency determinant (CFD) score for each off-target is also listed in the off-target annotation table, which is calculated using data from Doench *et al.*^3^ to estimate the likelihood of Cas9 targeting this sequence. Each mismatch position is assigned a value based on the change from one specific nucleotide to another as well as the position of the mismatch within the sgRNA sequence. These values are then multiplied, producing a number between 1 and 0, with 1 being more likely to cut at the off-target and zero being less likely. These scores can then be used to rank potential off-targets and filter sgRNAs based on their ability to produce off-target effects. *crispRdesignR* does not consider the position of the query target DNA sequence when finding possible off-targets so that the user may verify the location of their sgRNA target sequences within the genome in the off-target annotation table.

### Functions

All data is generated with a single function in R^17^: sgRNA_design(*userseq*, *genomename*, *gtfname*, *userPAM*, *calloffs* = TRUE, *annotateoffs* = TRUE).

- *userseq*: The target sequence with which to generate sgRNA guides - a character sequence containing DNA bases (A,C,T,G) or the name of a FASTA or plain text file in the working directory.
- *genomename*: The name of a genome (in BSgenome format) to check for off-targets and provide locations for sgRNA guides. These genomes can be downloaded through BSgenome or compiled by the user.
- *gtfname*: The name of a genome annotation file (.gtf) in the working directory to annotate sgRNAs and off-target sequences.
- *userPAM*: An optional argument used to set a custom PAM for the sgRNA. If not set, the function will default to the "NGG" PAM. Warning: the accuracy of efficiency scores has only been tested for the "NGG" PAM.
- *calloffs*: If TRUE, the function will search for off-targets in the genome chosen specified by the *genomename* argument. If FALSE, off-target calling will be skipped.
- *annotateoffs*: If TRUE, the function will provide annotations for the off-targets called using the genome annotation file specified by the *gtfname* argument. If FALSE, off-target annotation will be skipped.
- *getsgRNAdata*(x): This command is used to retrieve the data on the generated sgRNA sequences, where x is the raw data generated by *sgRNA_design*().
- *getofftargetdata*(x): This command is used to retrieve the additional off-target data, where x is the raw data generated by *sgRNA_design*().

*crispRdesignR* makes use of the R packages vtreat^13^ , gbm^12^, BSgenome^15^, BioStrings^16^, shiny^18^, stringr^19^ and DT^20^. Sequence homology features are calculated based on the sgRNA interaction screen reported in Thyme *et. al.* 8. The full list of commands can be found on the software webpage https://github.com/dylanbeeber/crispRdesignR.

## Results

**Figure 1:**
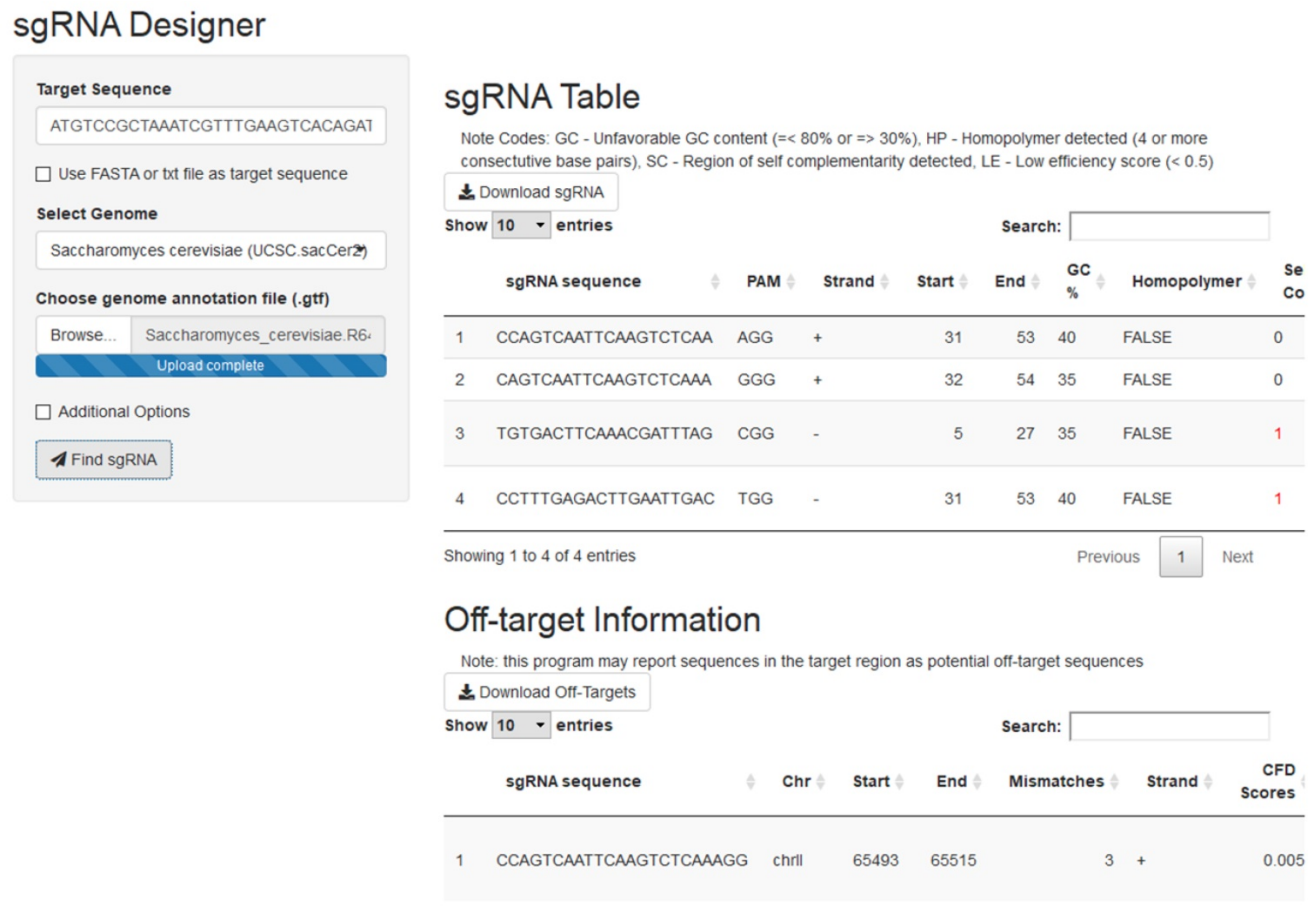
A screen capture from the *crispRdesignR* GUI demonstrating the target sequence, genome selection, and genome annotation file inputs. Partial sgRNA results and off-target annotations are also shown.

The *crispRdesignR* tool is built entirely in the R programming language, utilizing various packages to assist with different aspects of the program (see Materials and Methods). The program can be run on the command line or through a graphical user interface (GUI). Guide RNAs are designed based on a 23 base pair sequence from a user-input DNA sequence or FASTA file that ends with the PAM. The only hard limitation on DNA regions that can be used as guide RNA is the presence of the PAM site, 5'NGG3' in the case of SpCas9, the most commonly used Cas9 enzyme. In order to effectively provide a score for the experimentally-supported scoring method used in *crispRdesignR*, flanking sequence is also collected; this flanking sequence includes the four base pairs before the 5' end of the sgRNA and three base pairs after the 3' end of the PAM sequence. In total, a region of 30 base pairs is collected for each possible sgRNA. *crispRdesignR* exclusively designs 20-mer sgRNAs to keep the flanking sequence collected consistent with the Doench Rule Set 2 model^3^. The R package searches for sgRNAs from the input and returns a table listing candidate sgRNAs and their sequence features, and optionally returns annotated off-target information in a user-chosen genome (Figure 1). The GC content of each target sequence is calculated excluding the PAM site, as the GC content of the PAM does not affect binding to the target region^3^. The self-complementarity integer provided by *crispRdesignR* reports the number of possible 4-nucleotide regions of self-complementarity within both the sgRNA target sequence and the region on the sgRNA backbone that is prone to forming hairpins. Regions of self-complementarity between the target and itself as well as the target and the sgRNA backbone are given the same weight as both have been shown to greatly reduce activity^8^. Homopolymers are detected by searching for strings of 4 or more consecutive base pairs.

*crispRdesignR* has adopted the efficiency scoring method developed by Doench *et al.* (2016), employing a gradient boosted regression model trained on the FC and RES data sets^3^. In accordance with the Doench Rule Set 2, our model accounts for the presence of position-dependent single nucleotides, position-dependent dinucleotides, single nucleotide count, dinucleotide count, GC count, nucleotides that bookend the PAM sequence, and thermodynamic features of the target sequence plus context region (30-mer). The presence of specific nucleotides at certain positions in a sgRNA target site can influence the activity of that site. *crispRdesignR* considers the single and dinucleotides at each position and converts them into features that the machine learning model uses to predict activity.

To find off-target hits for the sgRNA, the genome from a user-selected species is loaded into *crispRdesignR* through the BSgenome package^15^, and each guide RNA is then searched through the genome for up to 4 mismatches. Once a complete list of matching sequences with genomic locations has been collected, the program then cross-references the matching locations with gene information provided in a user-input gene annotation file (.gtf). If the sgRNA matches a position in a gene, *crispRdesignR* reports the gene name as well as whether the match lies in a coding region.

**Figure 2:**
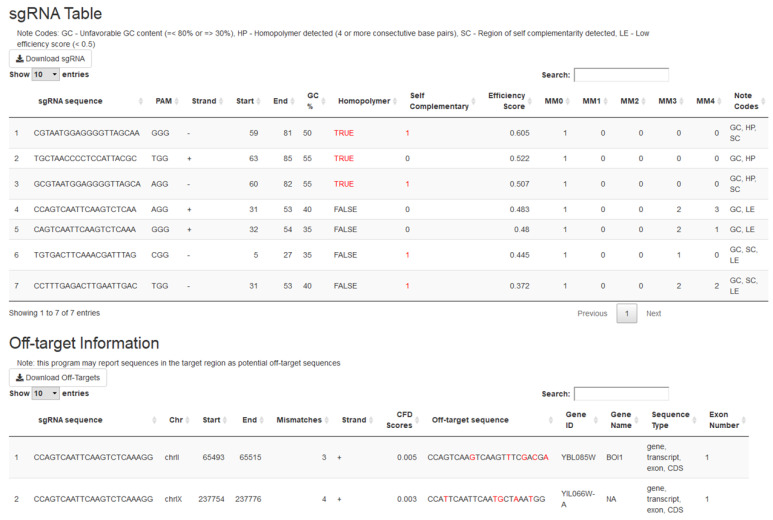
The output tables of *crispRdesignR* using a partial version of the DAK1 gene sequence, which is provided with the package download. Not all off-target matches are shown in the screenshot. Columns in the sgRNA table include sgRNA sequence, PAM, direction, start, end, GC content, presence of homopolymers, possible self-complementary sequences, efficiency score^3^, number of matches in the user-provided genome with between 0 and 4 mismatches (MM), and notes. The Off-target information table includes the original sgRNA sequence, chromosome, start, end, number of mismatches, strand, CFD scores, matched sequence, gene ID, gene name, sequence type, and exon number.

Running *crispRdesignR* will output two tables (Figure 2). The first table contains the information on each individual sgRNA, including the sequence, PAM, location, direction relative to the target sequence, GC content, homopolymer presence, self-complementarity, off-target matches, predicted efficiency score, and a column that summarizes unfavorable sequence features. The second table contains the information about each off-target match, including the original sgRNA, off-target sequence, chromosome, location, direction relative to the target sequence, number of mismatches, gene ID, gene name, genome feature, and exon number. These tables can be sorted and searched through the GUI or downloaded as .csv files for further analysis. The location of the original sgRNA target sequence in the genome can be found in the off-target information section for identity verification. If no genome is provided or off-target searching is skipped, no data will be provided in the off-target matches column or the off-target information table.

### Speed Comparisons

We compared the runtime of various sgRNA design programs based on what a user might experience. *crispRdesignR* has relatively fast runtimes to discover sgRNA sequences compared to other tools, although using custom genomes that are not pre-indexed leads to increased runtimes when choosing to call and annotate off-targets (Table 2). Most web-based programs have pre-indexed genomes for fast off-target calling, but indexing can take several hours to perform and as such is not always ideal for users uploading custom genomes or for few queries. On a desktop with 3.4 GHz CPU and 8.00 GB RAM, the run time for a 128 bp sequence (“DAK1 short”, provided with the program) in *S. cerevisiae* averages out to 8 seconds in *crispRdesignR* when calling off-targets (3 seconds without off-target calling) compared to 7 seconds in *CRISPOR*^21^ and 5 seconds in *CHOPCHOP* v2^22^,^23^. *GuideScan*^24^ has some of the shortest runtimes when genomic coordinates are known and provided beforehand (2-3 seconds in *H. sapiens* and *S. cerevisiae*), but the web application can take over a minute if provided a FASTA file when searching the human genome. *crispRdesignR* is comparable in terms of speed with another R package, *CRISPRseek*^25^*,* where* crispRdesignR* has a speed advantage when searching smaller genomes and *CRISPRseek* is faster with larger genomes. While not requiring genome indexing can save hours of run-time before conducting guide-design, the trade-off is that it extends the time of off-target searches: with the human genome, each additional sgRNA generated by *crispRdesignR* will add about 1 minute of run time. To reduce run-time when searching for off-targets, it is recommended that users keep DNA query sequences under 250 bases pairs when searching against a genome containing over a billion base pairs.

### Efficiency Score Validation and Ranking Comparisons

The sgRNA efficiency score ranking from *crispRdesignR* is largely consistent with other programs, with the four highest ranked sequences also found in the top five highest ranked sequences generated by most other programs tested (Table 3). Efficiency scores are expected to vary slightly from program to program as different applications are likely using variations of the predictive model described by Doench Rule Set 2. Scores generated by *crispRdesignR* have been tested against the human ribosomal, human non-ribosomal, and mouse all essential sgRNA data sets provided in Xu *et al.*^26^. These data sets contain sgRNAs designated as effective versus ineffective in both human and mouse cell lines. The scores provided by *crispRdesignR* clearly distinguish between effective and ineffective sgRNA in each of the three data sets (P-values < 0.001, two-sample Kolmogorov-Smirnov test), reflecting the results in Doench *et al.*^3^.

When evaluating scores against other currently available sgRNA design programs, *CHOPCHOP* and *CRISPOR* provide identical prediction rankings and efficiency scores (after rounding), whereas *crispRdesignR* and *GuideScan* provide identical prediction rankings with different efficiency scores. *crispRdesignR* additionally predicts a unique off-target with 3 mismatches in the 3^rd^ ranked guide sequence, which was not found by any of these other applications (Table 3). This possible off-target contains 3 mismatches within the target itself and 2 within the PAM; other applications might count this as 5 mismatches and not report it as an off-target. However, mismatches within the target sequence are not tolerated in the same ways as mismatches in the PAM^3^, and as such are treated as separate in the mismatch calculation used in *crispRdesignR*. Additionally, the PAM for this particular off-target does not match the “NGG” template but is an alternative PAM that has been found to be targeted by SpCas9 in some circumstances based on the CFD off-target scoring predictions^3^. These reasons may prevent the other applications from reporting this particular sequence as a potential off-target.

## Discussion

When utilizing web-based sgRNA design programs, a user is often limited by a list of preinstalled genomes. *crispRdesignR* sets itself apart by allowing the user to import a custom genome and/or genome annotation file to search for sgRNAs and off-target effects. The *crispRdesignR* software provides comprehensive sequence features to the user that are often omitted from other prominent sgRNA design programs (Table 1). The complete sequence feature information provided by *crispRdesignR* is very well-suited to applications where efficiency scores are of limited use. When using efficiency scoring methods with conditions that they have not been trained on (for example different organisms, cell types, or PAMs), the efficiency predictions will be less accurate. However, the predictive power of the model may not be completely lost if efficiency scoring methods are used in addition to known effects of various sequence features on activity to eliminate inactive sgRNA^3^.

The open source nature of *crispRdesignR* allows users to build on the features of the software for their specific uses. The gradient boosted regression model that *crispRdesignR* uses for efficiency scoring can be trained on other experimental data sets that contain the sgRNA sequence plus context (30-mer) and guide rankings assigned scores between 0 and 1. This allows for user-generated efficiency scoring models trained on data relevant to that user's needs. However, for this to be a strongly predictive model, activity data must be available and normalized for thousands of sgRNA sequences in that relevant context^3^. The accessibility of the output tables as .csv files generated by *crispRdesignR* also allow a user to easily isolate the sgRNA sequences and run them through other scoring models that are more appropriate for a specific application but that lack the sequence features, off-target annotation, or genome customization of *crispRdesignR*.

The flexibility and details that are provided by the robust off-target annotation system used by *crispRdesignR* currently limit the speed of the program. While other programs might allow a user to index genomes for quicker searching, the process of indexing a custom genome can be hardware intensive and overall slower than a few searches on an unindexed genome for off-targets, particularly for design applications in a small target region. For applications that require sgRNA design in a large target region (over 1000 base pairs) within a large genome (over 1 billion base pairs), the user can bypass off-target calling in *crispRdesignR* to prevent long run times. Although web-based programs that access pre-indexed genomes offer superior speed, we show that they often report less sequence feature information and they are limited to a pre-defined list of genomes. *CHOPCHOP* v2^22^,^23^ is one of the few applications that will provide the GC content of each sgRNA sequence, but it provides the GC content of both the target sequence plus the PAM site, instead of the target site alone (however, this has been corrected in the newer version of *CHOPCHOP* v3^27^). The web-based version of *CHOPCHOP* v3 allows handling large jobs and uncommon genomes through command-line scripts, but whereas added genomes must be submitted to the developers and approved before use, *crispRdesignR* allows custom genomes to be directly and immediately used via its R interface.

Another R package, *CRISPRseek*^23^, uses similar methods of efficiency scoring and off-target calling, allowing for searching custom genomes and annotation files. However, it lacks the graphical user interface and several sequence features provided by *crispRdesignR*. The two programs both take longer to run than many of their web-based counterparts due to their ability to use non-indexed genomes. Not requiring pre-indexed genome files allows* crispRdesignR* to be installed quickly and run on any custom genome, still performing relatively rapidly when designing a handful of guides or targeting small genomes. Although both *crispRdesignR* and *CRISPRseek* use the same efficiency scoring method, *CRISPRseek* requires the user to add python packages in order to obtain the scores based on Doench Rule Set 2^3^. *crispRdesignR* is able to provide scores based on Rule Set 2 completely within R. Each program contains exclusive features that the other lacks that may be useful in different settings. For example, *CRISPRseek* has the ability to filter sgRNA based on restriction enzyme cutting sites, while *crispRdesignR* detects possible self-complementary sgRNA sequences.

The R package *crispRdesignR* sets itself apart by allowing the user to import a custom genome and/or genome annotation file to search for sgRNAs and off-target effects, while providing extensive target sequence information and the option of an accessible GUI. These unique features make *crispRdesignR* particularly useful for non-bioinformaticians working with uncommon organisms, non-standard cell types, and alternate PAMs. *crispRdesignR* allows users to seamlessly integrate data from other R-based workflows, including accessing and formatting additional genomes through BSgenome^15^. Accessible source code provides transparency and further adds to the versatility of *crispRdesignR* as efficiency scoring methods are developed and future technological improvements are made.

## Figures and Tables

**Table 1:** Feature comparisons between several prominent free sgRNA design programs *CHOPCHOP* v2^22^,^23^, *CRISPR Design*^3^, *CRISPRseek*^25^, *CRISPOR*^21^, and *GuideScan*^24^. Features reported include whether all targets that match the PAM are output (All targets), the scoring method from Doench^3^, Moreno-Mateos^4^, or customizable), self-complementarity through hairpin detection, GC content, homopolymer filtering, the maximum number of mismatches permitted between the guide sequence and reference, the available PAM sequence, and whether off-target sequences are reported and annotated.

Software name	*CHOPCHOP v2*^22,23^	*CRISPR Design*^3^	*CRISPRseek*^25^	*CRISPOR*^21^	*GuideScan*^24^	*crispRdesignR*
Providing entity	Harvard	Broad Institute	UMASS Medical	Tefor	MSKCC	UML
All targets	Yes	No	Yes	Yes	Yes	Yes
Scoring method	Customizable	Doench	Doench	Doench & M.-Mateos	Doench	Doench
Hairpins	Yes	No	No	No	No	Yes
GC content	Yes	No	No	No	No	Yes
Homopolymers	No	No	No	No	No	Yes
Max no. of mismatches	3	4	4	4	3	4
PAM	Customizable	NGG, NNGRR	Customizable	Customizable	NGG, TTTN	Customizable
Off-target Annotation	No	Limited	Yes	Yes	No	Yes

**Table 2:** Runtime comparisons for example sequences in each program analyzed. Run times (minutes:seconds) were averaged over three trials on a desktop PC (windows 10 OS, 3.4 GHz CPU with 4 cores, solid state drive, and 8.00 GB RAM). Some programs offered a limited list of available genomes that prevented analysis (indicated by N/A). Test sequences were picked to showcase a variety of sequences that guides may be designed for and each was searched for off-targets throughout the target organism's whole genome. The DAK1 short example sequence can be found on the *crispRdesignR* github site; it is 128 bp long and generates 13 target sequences, with 35 off-targets. The DAK1 sequence contains 1780 bp and generates 170 target sequences, with 495 off-targets. The MYBPC3 deletion sequence contains 57 bp and generates 6 target sequences, with 2,219 off-targets. The Partial ADRB1 sequence contains 70 bp and generates 11 target sequences, with 9,200 off-targets.

Test Sequence	Genome	*CHOP-CHOP*^22,23^	*CRISPR Design*^3^	*CRISPRseek*^25^	*CRISP-OR*^21^	*GuideScan*^24^	*crispRdesignR* (no off-targets)	*crispRdesignR* (with off-target calling)
DAK1 short	*S. cerevisiae* (yeast)	0:05	N/A	2:10	0:07	0:02	0:03	0:08
DAK1	*S. cerevisiae* (yeast)	0:18	N/A	4:24	0:19	0:02	0:14	1:47
MYBPC3 deletion	*H. sapiens* (human)	0:06	0:15	6:50	0:10	0:03	0:03	7:36
Partial ADRB1	*H. sapiens* (human)	0:34	0:26	14:35	0:15	0:03	0:05	15:42

**Table 3:** Comparison of efficiency scores of sgRNAs for the DAK1 short sequence from five different sgRNA design applications: *CHOPCHOP*, *CRISPRseek*, *CRISPOR*, *GuideScan* and *crispRdesignR*. Only the efficiency scores for the top 5 guide sequences from each program are shown.

Guide Sequence	*CHOPCHOP*^22,23^	*CRISPRseek*^25^	*CRISPOR*^21^	*GuideScan*^24^	*crispRdesignR*
CAGGGACCAGCGTAATGGAG	63.75		64	68	0.660
CGTAATGGAGGGGTTAGCAA	64.17	0.728	64	63	0.605
TCAGGGACCAGCGTAATGGA	57.25	0.623	57	59	0.575
TGCTAACCCCTCCATTACGC	58.74	0.283	59	56	0.522
GCGTAATGGAGGGGTTAGCA				56	0.507
TTCAGGGACCAGCGTAATGG	56.3		56		
TGTGACTTCAAACGATTTAG		0.577			
CAGTCAATTCAAGTCTCAAA		0.321			
